# Anesthesia for Awake Craniotomy for Brain Tumors in an Intraoperative MRI Suite: Challenges and Evidence

**DOI:** 10.3389/fonc.2018.00519

**Published:** 2018-11-14

**Authors:** Tumul Chowdhury, Gyaninder P. Singh, Frederick A. Zeiler, Abseret Hailu, Hal Loewen, Bernhard Schaller, Ronald B. Cappellani, Michael West

**Affiliations:** ^1^Department of Anesthesiology, Perioperative and Pain Medicine, University of Manitoba, Winnipeg, MB, Canada; ^2^Department of Neuroanaesthesiology & Critical Care, Neurosciences Centre, All India Institute of Medical Sciences, New Delhi, India; ^3^Section-Neurosurgery, Department of Surgery, University of Manitoba, Winnipeg, MB, Canada; ^4^Clincian Investigator Program, University of Manitoba, Winnipeg, MB, Canada; ^5^Division of Anaesthesia, Department of Medicine, Addenbrooke's Hospital, University of Cambridge, Cambridge, United Kingdom; ^6^Max Rady College of Medicine, University of Manitoba, Winnipeg, MB, Canada; ^7^College of Rehabilitation Sciences, Neil John Maclean Health Science Library, University of Manitoba, Winnipeg, MB, Canada; ^8^Department of Primary Care, University of Zurich, Zurich, Switzerland

**Keywords:** anesthesia, awake craniotomy, intraoperative MRI, brain tumor, safety & hazards

Awake craniotomy allows for continuous monitoring of neurological functions during the surgery for brain tumors at or near the eloquent areas. It enables monitoring of language and motor functions by communicating with the patient and observing the patient during speech and motor tasks (1, 2). With the technological advancements over the years, intraoperative magnetic resonance imaging (I-MRI) has emerged as a promising technique to guide the intraoperative resection of brain tumors. It offers the advantage of providing real-time intraoperative MRI images that detect tumor remnants as well as improves accurate anatomical localization by compensating for the brain shift that occurs during the surgery (3–5). In various studies, I-MRI has shown to improve and maximize resection of lesions thereby improving survival (6, 7). However, use of I-MRI alone does not delineate safe boundaries for a resection nor does it reliably preserve the neurological functions (8).

Thus, the combination of the awake craniotomy and I-MRI has been used to optimize maximize safe resection taking advantage of each technique. The two techniques when used simultaneously seem to supplement each other by attaining maximal resection but minimizing neurological dysfunction. However, each of these techniques has specific concerns and challenges and using both together makes the procedure further demanding. This opinion based article presents anesthetic considerations based on current evidence from the analysis of secondary outcomes (anesthetic) in our systematic review (ROSPERO, International prospective register of systematic reviews, CRD42016052733) and is complementary to a recently published systematic review on the topic of awake craniotomy techniques during I-MRI (9).

## Challenges during I-MRI

An I-MRI operating room posses a unique environment that produces several new concerns for the physicians which include restrictions on the use of ferromagnetic equipment because of the high magnetic field, the use of extra-long breathing circuits, long intravenous line tubing, and monitoring cables (required for movement of the patient in and out of MRI gantry). Moreover, there are other important concerns including hypothermia due to the low temperature in the MRI suites, loud acoustic noise with the potential to cause hearing problems, and other patient-specific concerns (implanted pacemaker, defibrillators, ferromagnetic metallic implants, and aneurysmal clips), which require proper patient screening and selection (10). There is a constant high magnetic field in the I-MRI room, which necessitates the use of only MRI compatible equipment and devices beyond the 5 Gauss line. Further, a direct contact with any conductive material like ECG leads, radiofrequency (RF) coils, may cause burns to the patient due to induction currents and RF heating (10).

## Challenges during awake craniotomy

Success of awake craniotomy procedures essentially depends on the patient's cooperation. A complaint and a motivated patient is required for continuous neurological testing during the awake craniotomy. A thorough evaluation before the surgery is important to explain the rationale, and technique to the patient in order to achieve better patient cooperation during the surgery. Also, a complete evaluation of an airway is essential to identify patients with a potentially difficult airway. Complexity of an awake craniotomy is increased in a patient with a difficult airway or history of severe obstructive sleep apnea. Such patients may not be appropriate candidates for this procedure. Moreover, the patient has to remain in one position for a considerable period of time, and this sometimes leads to fatigue.

Thus, concerns about the patient's airway control, physical constraints, prolonged duration of surgeries, proper patient positioning, patient's comfort and safety, intraoperative thermoregulation, repeated intraoperative imaging with multiple transfers of the patient in and out of the MRI scanner, complex operative setup and equipment restrictions make the combination of these two techniques a potentially challenging task.

We reviewed the available literature for anesthetic management during neurosurgical procedures, which were performed under awake craniotomy with I-MRI (1, 2, 8, 11–17). Though both awake craniotomy and I-MRI have been frequently used in isolation to guide brain surgeries near the eloquent areas of brain, there is limited literature on the combined use of both of these techniques in such surgeries and authors have reported their experience in only a small number of patients. Moreover, the strength of the magnetic field varied in different studies (Table 1).

**Table 1:** Anesthesia techniques during combination of the awake craniotomy with I-MRI.

**References**	**Cases (n)**	**I-MRI (Magnet/field)**	LA drug^*^	**Anesthesia technique**	**Airway device used**	Anesthetic drug^**^
Nabavi et al. (12)	38	Fix/1.5T	Rop (2.5%)	LA + Sedation (MAC)	No	P + R
Weingarten et al. (14)	10	Fix/1.5T	ND	LA + Sedation (MAC)	No	NA
Goebel et al. (13)	25	Fix/1.5T	Bup (0.375%)	LA + Sedation (MAC)	No	P
Leuthardt et al. (11)	12	Mov/1.5T	ND	LA + AWA	LMA	P + D + A
Lu et al. (8)	30	Mov/3T	Lido (0.67%) + Rop (0.5%)	LA + Sedation (MAC)	No	M + D + R + P
Tuominen et al. (1)	20	Fix/0.23T (open scanner)	Bup + Lido	LA + Sedation (MAC)	No	P + F
Maldaun et al. (2)	42	Fix/1.5T	Rop (0.5%) + Lido (1%) + Bup (0.25%)	LA + AWA	LMA	P + R + D + S + I
Zhuang et al. (17)	20	Mov/3T	Lido (0.67%) + Rop (0.5%)	LA + Sedation (MAC)	No	M + D + R + P
Coburger et al. (15)	9	ND/0.2T (open scanner)	ND	ND	ND	ND
	17	ND/1.5T	ND	ND	ND	ND
Ghinda et al. (16)	106	Mov/3T	ND	LA + Sedation (MAC)	No	P + D + R

* *For Scalp Block, dural surface, pin and incision site infiltration*,

** *For sedation/Analgesia*.

*n, number of procedures, T, tesla, I-MRI, intraoperative magnetic resonance imaging, Fix, fixed magnet; Mov, movable magnet, LA, local anesthesia; P, propofol; R, remifentanil; D, dexmedetomidine; A, alfentanil; M, midazolam; F, fentanyl; S, sevoflurane; I, isoflurane; Rop, ropivacaine; Bup, bupivacaine; Lido, lidocaine; AWA, asleep-wake-asleep; LMA, laryngeal mask airway; ND, no data*.

## Selection of patients

Based on current literature, it is unclear as to what percentage of patients undergoing craniotomy for tumors are eligible for awake craniotomy during I-MRI, as the selection of such patients is multi-factorial. Such decisions are based on tumor location, expected duration of procedure, patient's pre-operative clinical status, ability to cooperate, and experience of the operative team. It cannot be overemphasized the need for well-informed cooperative patients. Much of the success of such procedures rely heavily on the pre-operative discussion, ensuring that an accurate depiction of the procedure and operative timeline are portrayed. Little information is provided in the literature regarding these pre-operative discussions. At our institution, it is not uncommon to have multiple lengthy visits between the surgical team and the patient/family to discuss the details of such an operation prior to scheduling the procedure.

In general, the following aspects of the patient selection should be considered. Patients harboring lesions near or within the eloquent areas (motor, language, vision) are subjected to awake craniotomies along with I-MRI. Patients are evaluated for their eligibility to undergo awake craniotomy with regards to their physical, mental (cognitive) and affective status. Patients with major neurological deficits that preclude testing of function (pronounced aphasia or motor), poor neuropsychological scores (Mini-Mental State Examination, Aphasia Quotient Score for language function), significant deficits in concentration and sustained attention, patients suffering with severe anxiety or depression, claustrophobia, disinhibition, apathy and disorganized behavior are not candidates for awake craniotomy. Patients found eligible for awake craniotomy are informed about the intraoperative motor, sensory and language testing and are encouraged to rehearse this testing in the preoperative period. Moreover, patients are assessed for any contraindication to undergoing the procedure in a magnetic environment.

## Techniques of awake craniotomy with I-MRI

At different centers awake craniotomy is performed either as an asleep-awake-asleep or awake-awake-awake technique with or without sedation under monitored anesthesia care (MAC). In the asleep-awake-asleep technique, the patient is anesthetized throughout the procedure except during monitoring of neurological function. The patient is awakened for neurological function monitoring during surgery and again anesthetized once the resection is complete and neurological monitoring is no longer required. In contrast to this, during the awake-awake-awake technique, the patient remains awake throughout the surgery. In most of these cases, conscious sedation is used to make the patient comfortable during these long procedures and the patient is monitored for their vital signs, neurological status, and level of sedation (MAC). Given the complex environment of the I-MRI suite, the duration of anesthesia is also prolonged substantially. This is attributed by many factors including performing anesthetic counts (depending upon scan frequencies), number of scans, further resection, setting up specialized infusion pumps, applying monitors, establishing invasive lines with extra-connections and length, positioning of the patient (both for the operation and scans), changing the anesthetic techniques (sedation to general anesthesia depending upon the patient and institutional preference for the scan period), and managing intraoperative complications (seizures, airway compromise), if any.

Before the start of the procedure, the standard MRI-compatible monitors are attached. Some authors use asleep-awake-asleep technique and patients are anesthetized using anesthetic and analgesic drugs, with the airway secured using LMA during the initial phase of surgery (2, 11). Other authors utilize a complete awake technique with the patient remaining awake throughout the surgery, breathing spontaneously. Oxygen is supplemented using nasal cannula or facemask. In all patients, adequate intravenous access (peripheral and/or central venous catheter) is obtained and both an arterial line and urethral catheter are placed.

For an awake craniotomy, a scalp block is performed by infiltrating with the local anesthetic. Sedation is usually administered prior to the scalp block to make the procedure comfortable for the patient. After an adequate level of sedation is achieved, the injection of local anesthetic drug (lidocaine, bupivacaine or ropivacaine) is given to block the nerves supplying the scalp. Various nerves supplying the scalp (supratrochlear, supraorbital, zygomaticotemporal, auriculotemporal, lesser and greater occipital nerves) are blocked on either side or a ring block is performed. The level of sedation is monitored using various monitors of sedation (Bi-spectral index, entropy) to maintain an adequate level. Drugs commonly used for sedation are propofol, fentanyl, remifentanil, alfentanil, midazolam, and dexmedetomidine (Table 1). In contrast to other drugs, dexmedetomidine produces sedation without causing respiratory depression.

After the scalp block, patients are positioned for surgery with adequate padding (ensuring maximal comfort). The pin sites are infiltrated with local anesthetic and the head is fixed to the table with a modified high-field MR imaging-compatible head holder. The surgical drapes are arranged in an “open tent fashion” such that the face is not covered under the drapes. It reduces anxiety and discomfort to the patient and allows direct eye contact and face-to-face conversation with the patient during the neurological testing. Oxygen is administered through nasal cannula or facemask during the surgery. Since the ambient temperature of the I-MRI operating room is low, hypothermia is prevented by covering the patient with drapes and use of warming devices placed outside the 5 Gauss line. To minimize brain swelling caused by higher CO_2_ levels associated with awake craniotomy (non-intubated and sedated patients), a diuretic is given intravenously (mannitol, frusemide) at the start of surgery. Elevation of the head may further help reduce brain swelling.

Before incision, the site of skin incision and the base of skin flap are infiltrated with local anesthetic. The next potential pain sensitive structure encountered is the dura mater. Since manipulation of the dura mater and large vessels can elicit pain, after removing the bone flap, cottonoids immersed in the local anesthetic are placed over the dura mater (12). During the opening of the cranium, additional doses of opioids may be infused if required. Throughout the surgery, the neurosurgeon, neuropsychologist/neurophysiologist and anesthesiologist maintain verbal contact with the patient. In the asleep-awake-asleep technique the patient is awakened during the surgery for neurological testing. The anesthetic drugs are stopped and the airway device (LMA) removed once the patient is fully awake. The oxygen is supplemented through nasal cannula or face mask. Overall, surgical times are not increased, given the technique prior to I-MRI is identical to that utilized for awake craniotomy procedures in the absence of I-MRI. The only caveat to this comes from the results of the I-MRI, where surgical time may increase if it is elected to continue resection given the presence of residual accessible disease.

Once the tumor resection, guided with clinical testing of neurological function in the awake patient, is complete and I-MRI is planned, preparation and transfer to the imaging unit is required. The added duration that the I-MRI component contributes to the procedure varies depending on the particular MRI system utilized and local safety protocols (i.e., instrument counting and safety checklists), with our recent systematic review on the topic suggesting upwards of 75 min for scan time alone (9). Our personal experience finds that the I-MRI (i.e., protocols and scan time) adds ~2–3 h to the overall operative time. Preparation includes covering the operation site with sterile drapes, checking and removal of any ferromagnetic object including needles (to prevent entry of such object beyond the 5 Gauss line), avoid traction and pulling of extra-long lines and catheters (oxygen tubing, arterial line, intravenous fluid and infusion tubing), and prevent coiling of monitor cables and wires. Earplugs are placed in the patient during imaging for noise protection. During I-MRI scanning, the patient is covered with sterile drapes. Either the operating site is completely covered with the sterile drape with the face uncovered and a high flow of oxygen is administered through a nasal cannula (8, 12, 13) or the patients are wrapped in a sterile full-body non-breathable drape (11). In the latter case, there is no access to the patient's airway and it takes a few minutes to withdraw the patients from MR gantry and extricate the patient from the drapes. Thus, an LMA or endotracheal tube may be used to maintain a safe airway during the acquisition of images (11). After the I-MRI imaging, the patient is again shifted out of the MRI unit to the operating area. Depending on the findings of the I-MRI scan, the further resection of residual lesion or closure of the craniotomy is decided. In cases where no further awake neurological clinical assessment is required, the patient is either sedated (MAC) or anesthetized and airway secured (asleep-awake-asleep) during the closure to make the procedure comfortable for the patient.

Finally, any adverse event that may occur during the surgery needs prompt management. Focal seizures are usually managed by irrigating the cortex with ice-cold saline/ringer lactate, without the need for intravenous medication. However, generalized seizures may require the administration of an antiepileptic agent. Rarely, loss of airway due to oversedation or during the postictal phase may lead to desaturation and inadequate ventilation with a rise in CO_2_ levels and brain swelling. This may require securing of the airway with supraglottic airway devices or endotracheal tube. Other cardiovascular events, such as hypotension, hypertension, tachycardia, bradycardia or other arrhythmias need to be managed with appropriate drugs and/or fluids.

## Limitation

As such, this article was designed to provide an opinion based overview of the anesthetic considerations involved in awake craniotomy techniques during I-MRI. It was not designed to be either completely comprehensive in its opinionated overview, nor present new and groundbreaking information, as such opinions should be based on the current evidence. Subsequently, the views presented are based on personal beliefs molded from both personal experience and the available body of literature till predefined date.

## Conclusion

Combining awake craniotomy and I-MRI offers the potential to improve the extent of safe resection for lesions located in close proximity to eloquent areas. Nonetheless, performing the surgical procedure under awake craniotomy with I-MRI is strenuous to the patients as well as the OR team. Patients should be carefully selected and extensively informed and counseled prior to the procedure. It requires a continuous motivation and cooperation of the patient while they are repetitively being asked to perform tiring tasks. Moreover, intraoperative imaging significantly adds to the total procedure time depending on the imaging sequences and number of times that imaging is performed. In spite of these multiple challenges faced by the anesthesiologists while providing anesthesia care to patients undergoing awake craniotomy combined with I-MRI imaging, anesthesia for these procedures can be safely performed.

## Disclosure

TC served as a guest associate editor for Frontiers in Neurosciences and is presently serving as an associate editor for Intensive Care Medicine and Anesthesiology section in Frontiers in Medicine. FZ has received salary support for dedicated research time, during which this manuscript was completed. Such salary support came from: the Cambridge Commonwealth Trust Scholarship, the Royal College of Surgeons of Canada—Harry S. Morton Traveling Fellowship in Surgery and the University of Manitoba Clinician Investigator Program. FZ's research is also supported through the Thorlakson Chair in Surgical Research Establishment Grant. BS served as a guest associate editor for Frontiers in Neurosciences.

## Author contributions

TC has developed the hypothesis, assisted substantially in data collection, screening, reviewing, analyzing, contacting the authors, compiling and writing the manuscript. GS has collected and screened the data and helped writing the manuscript. FZ has reviewed and screened the data, and helped substantially in writing the manuscript. AH has assisted in collecting the data. HL has provided the initial data, abstracts, full-texts and formulated the search tables/prisma flow chart. BS has assisted in writing, reviewing and editing the manuscript. RC has assisted in developing the hypothesis, reviewing and editing the manuscript. MW has assisted in writing/editing the manuscript.

### Conflict of interest statement

The authors declare that the research was conducted in the absence of any commercial or financial relationships that could be construed as a potential conflict of interest.
